# BIKSWITCH Study: Effectiveness and Safety of Switching to Bictegravir/Emtricitabine/Tenofovir Alafenamide (B/F/TAF) From Therapies Not Based on Integrase Inhibitors in Virologically Suppressed HIV‐Infected Patients

**DOI:** 10.1155/arat/8818830

**Published:** 2025-12-12

**Authors:** Fernando Pérez-Calvo, Francisco Jover-Diaz, Elisabet Delgado-Sánchez, Jorge Peris-García, María Ángeles Bernabéu-Martínez

**Affiliations:** ^1^ Clinical Medicine Department, Miguel Hernández de Elche University, Alicante, Spain; ^2^ Infectious Diseases Department, Hospital Clínico Universitario San Juan de Alicante, Alicante, Spain; ^3^ Pharmacy Department, Hospital Clínico Universitario San Juan de Alicante, Alicante, Spain

**Keywords:** bictegravir, highly active antiretroviral therapy, HIV infection, treatment switch, virological suppression

## Abstract

**Objectives:**

Beyond initial antiretroviral therapy (ART), bictegravir/emtricitabine/tenofovir alafenamide 50/200/25 mg (B/F/TAF) has been evaluated as an option for the continuation of treatment for virologically suppressed individuals who switch from other regimens. While treatment‐emergent resistance to bictegravir has not been observed in clinical trials, rare case reports have described resistance development during B/F/TAF therapy, typically in the context of suboptimal adherence. Its favourable safety profile, minimal drug–drug interactions and proven virological efficacy suggest that virologically suppressed individuals may benefit from switching to B/F/TAF.

**Methods:**

Descriptive, retrospective, observational study of adults with HIV infection receiving effective non‐INSTI (integrase strand transfer inhibitors)‐based ART who switched to a B/F/TAF, but not all were suppressed. We compared individuals’ viral loads, CD4+ lymphocyte counts, glomerular filtration rates, and lipid profiles before the switch and after 24 weeks of B/F/TAF. Safety was also assessed.

**Results:**

Seventy‐nine individuals switched from non‐INSTI‐based therapies to B/F/TAF. At 24 weeks after the switch, HIV‐1 RNA < 50 copies/mL was reported in 96.1% and 92.4% of participants per protocol and intention‐to‐treat analysis, respectively, compared to 81.0% at baseline. This represents a 15.1% increase in virologic suppression (viral load < 50 copies/mL) compared to baseline. No significant changes were detected in the CD4+ lymphocyte count, glomerular filtration rate, or lipid profile. Adverse events (AEs) were reported in 5.1% of patients. Three patients discontinued treatment due to AEs.

**Conclusions:**

The results of this study suggest that patients can switch to B/F/TAF from non‐INSTI‐based therapies without compromising the effectiveness or safety of ART.

## 1. Background

Bictegravir, an integrase strand transfer inhibitor (INSTI), has emerged as a key component of modern antiretroviral therapy (ART) for people living with HIV. Co‐formulated with emtricitabine and tenofovir alafenamide (TAF) as a single‐tablet regimen (B/F/TAF), it is now recommended as a first‐line therapy in major international guidelines, including those from Europe and the United States [1–3]. The widespread adoption of B/F/TAF is supported by some pivotal randomized trials (GS‐US‐380‐1489, GS‐US‐380‐1490, GS‐US‐380‐1844, GS‐US‐380‐1878) for high virological efficacy, favourable safety and tolerability profile, minimal drug–drug interactions, and high genetic barrier to resistance [4–7]. Additionally, its once‐daily, single‐tablet formulation is associated with improved adherence, which is a critical factor in achieving and maintaining virological suppression in the long term.

Beyond initial ART, B/F/TAF (50/200/25 mg) has been evaluated as an option for the continuation of treatment for virologically suppressed individuals who switch from other regimens [7–9]. Clinical trials have demonstrated solid evidence that B/F/TAF is noninferior in terms of maintenance of virological suppression when compared with regimens based on other INSTIs, such as dolutegravir [10, 11]. In addition, bictegravir has demonstrated efficacy even in patients with documented primary resistance to first‐generation INSTIs (raltegravir, elvitegravir) who can maintain effective virological suppression regardless of the history of therapeutic failure with other ARTs, although there is the caveat about small sample size [12]. Several phase 3 clinical trials have demonstrated that switching to B/F/TAF is effective and well‐tolerated in virologically suppressed individuals, including switches from dolutegravir‐based regimens [7, 8], protease inhibitor‐based regimens, and other antiretroviral therapies.

Evidence from observational studies conducted in different cohorts [13–15] corroborates the findings of clinical trials. These findings support B/F/TAF as a preferred switch option in virologically suppressed patients. Despite widespread adoption and supporting data, additional clinical evidence is needed on the effectiveness and safety of B/F/TAF in patients switching from non‐INSTI regimens. These patients remain underrepresented in most studies, despite their prevalence in current treatment guidelines [1–3, 16].

## 2. Methods

### 2.1. Study Design and objectives

This retrospective observational cohort study was conducted at the San Juan University Clinical Hospital (HCUSJ) in Alicante, Spain. We included virologically suppressed adults living with HIV‐1 who switched from non‐INSTI‐containing ART regimens to B/F/TAF between May 2019 and July 2022. Inclusion criteria were age ≥ 18 years, documented viral suppression (HIV‐1 RNA < 50 copies/mL) for at least six months prior to switch, and availability of follow‐up data for at least 24 weeks postswitch. Patients with documented INSTI resistance or incomplete clinical data were excluded. Participants were followed until regimen change due to treatment failure or intolerance, loss to follow‐up, or study end.

Demographic and clinical data, treatment history, reasons for switching, laboratory parameters including HIV‐1 RNA viral load, CD4+ lymphocyte count, lipid profile, adverse events (AEs), and treatment discontinuations were obtained from electronic medical records with pseudo‐anonymization.

Virologic suppression was defined as plasma HIV‐1 RNA < 50 copies/mL. Viral loads were measured using the Abbott RealTime HIV‐1 assay with a lower limit of quantitation of 20 copies/mL; values below this threshold were classified as undetectable for analysis. Virological failure (VF) was defined as HIV‐1 RNA > 200 copies/mL after 24 weeks of ART.

The primary objective was to assess the effectiveness of switching to B/F/TAF by the proportion maintaining virological suppression at 24 weeks. Secondary objectives included evaluation of AEs, changes in CD4+ lymphocyte counts, glomerular filtration rate (estimated via CKD‐EPI), and lipid profiles (total cholesterol, HDL, LDL, and atherogenic index calculated as Castelli index). Intention‐to‐treat (ITT) analyses included all participants regardless of treatment completion; per‐protocol analyses included only those completing 24 weeks of B/F/TAF.

### 2.2. Statistical Analysis

Baseline characteristics are presented using descriptive statistics, including proportions. Qualitative variables are presented as percentages, and quantitative variables are presented as medians and interquartile ranges (IQRs) for non‐normally distributed variables and as means ± standard deviation for normally distributed variables. The Student’s *t*‐test and the Mann–Whitney test were used to compare continuous variables. The McNemar test was used for the comparison of categorical variables. Kaplan–Meier time to maintain virological suppression analysis was performed to estimate the time to maintain virological suppression, defined as plasma HIV‐1 RNA < 50 copies/mL. All statistical analyses were performed with SPSS 22.0 for Windows (SPSS, Chicago, IL, USA).

## 3. Results

### 3.1. Patient Characteristics

At the end of 2022, HCUSJ provided health coverage to 237,467 individuals, with 394 hospital beds. In the Infectious Diseases Unit, there were 772 adults with HIV infection undergoing follow‐up. Among these individuals, 380 (49.2%) were treated with the B/F/TAF combination. A total of 79 individuals who switched to B/F/TAF from non‐INSTI‐based therapies were included in the study. Of these, two did not complete 24 weeks of treatment, and one did not have enough clinical information due to loss of follow‐up. Detailed baseline characteristics of the study population are shown in Table 1.

**Table 1:** Baseline characteristics of patients.

	All patients (*N* = 79)
Age, median (IQR), years	54 (43–59)
Sex, *n* (%)	
Males	66 (83.5)
Females	13 (16.5)
Region, *n* (%)	
Spain	51 (64.5)
South America	21 (26.6)
Africa	3 (3.8)
Eastern Europe	2 (2.5)
Western Europe	1 (1.3)
Asia	1 (1.3)
HIV diagnosis period, *n* (%)	
Pre‐HAART era (1985–2007)	39 (49.3)
HAART era (2008–2014)	21 (26.6)
ART era (2015–present)	18 (22.8)
Unknown	1 (1.3)
No. of previous ART lines, median (IQR)	1 (1‐2)
No. of prior ART tablets, median (IQR)	1 (1‐2)
ART prior to switch, *n* (%)	
Based on NNRTI	50 (63.3)
Based on boosted PI	28 (35.4)
Combination of both	1 (1.3)
Rationale for switch, *n* (%)	
Adaptation to guidelines	23 (29.1)
Drug interactions	22 (27.8)
Previous ART intolerance	21 (26.6)
No. or size of tablets	7 (8.9)
Cardiovascular risk	4 (5.1)
Genetic barrier	2 (2.5)

Abbreviations: ART = antiretroviral treatment, HAART = highly active antiretroviral treatment, IQR = interquartile range, NNRTI = non‐nucleoside reverse transcriptase inhibitor, No. = number, OD = once daily, PI = protease inhibitor.

At baseline, 71 patients (89.9%) were receiving a tenofovir‐containing regimen: 32 (40.5%) with TAF and 39 (49.4%) with tenofovir disoproxil fumarate (TDF) prior to switching to B/F/TAF. Before switching, both the median number of ART lines received and the median number of ART tablets received were 1 (IQR 1‐2). At baseline, 19/79 patients (24Y.Y%) were on multitablet regimens.

Before switching to B/F/TAF, 63.3% of participants received regimens based on non‐nucleoside reverse transcriptase inhibitors, including 29.1% who received *efavirenz/emtricitabine/TDF*‐based regimens. A total of 35.4% of the participants received regimens based on boosted protease inhibitors, with darunavir/cobicistat/TDF/emtricitabine being the main regimen (29.1%).

The three most common reasons for switching to B/F/TAF were adaptation to therapeutic guidelines (29.1%), interactions with other drugs (27.8%), and intolerance to previous ART regimens (26.6%).

### 3.2. Efficacy Outcomes at Week 24

Regarding the primary objective, the number of participants with plasma HIV‐1 RNA levels < 50 copies/mL increased significantly, from 81.0% at baseline to 92.4% at Week 24 in the ITT analysis (96.1% in the per‐protocol analysis; *p* ≤ 0.013; Table 2). In the per‐protocol analysis, this represented a difference of 15.1%. Of the six patients who did not achieve undetectable levels of HIV‐1 RNA, one had a viral load < 200 copies/mL, and the other three did not complete 24 weeks of treatment or had no clinical information available. In two cases (2.6%), VF was detected and resistance test was performed. No resistance mutations had been detected. Among the two participants who experienced VF, clinical records indicated documented suboptimal adherence, including missed clinic visits and patient self‐reported nonadherence to the prescribed regimen. Importantly, both patients’ viral loads subsequently resuppressed without changes to their antiretroviral regimen.

**Table 2:** Changes in analytical characteristics after 24 weeks of treatment with B/F/TAF.

Variables	Baseline (*n* = 79)	Week 24 (*n* = 76)	*p* value
HIV‐1 RNA < 50 copies/mL, *n* (%)	64 (81.0)	73 (96.1)	0.013

HIV‐1 RNA ≥ 50 copies/mL, *n* (%)	15 (18.9)	0	≤ 0.001

HIV‐1 RNA in patients with ≥ 50 copies/mL at baseline, median (IQR)	74 (54–102)	21 (19–26)	< 0.001

CD4+ lymphocytes, median (IQR), cells/μL	*n* = 78	*n* = 74	> 0.06
	747.5 (497.5–1028.2)	747 (572–1039)	

TC, median (IQR), mg/dL	*n* = 77	*n* = 70	> 0.1
	196 (163–215)	179 (157.7–214)	

HDL‐C, median (IQR), mg/dL	*n* = 74	*n* = 60	> 0.2
	46.5 (40.2–53.7)	45.5 (38.2–53)	

LDL‐C, median (IQR), mg/dL	*n* = 61	*n* = 59	> 0.1
	119 (93.5–134)	103 (91–123)	

Atherogenic index, median (IQR)	*n* = 64	*n* = 60	> 0.6
	3.9 (3.1–4.6)	3.8 (3.1–4.5)	

No. of ART tablets, median (IQR)	*n* = 79	*n* = 76	≤ 0.001
	1 (1‐2)	1 (1‐1)	

Abbreviations: ART = antiretroviral treatment, B/F/TAF = bictegravir/emtricitabine/tenofovir alafenamide, HDL‐C = high‐density lipoprotein cholesterol, IQR = interquartile range, LDL‐C = low‐density lipoprotein cholesterol, No. = number, TC = total cholesterol.

At the time of switching to B/F/TAF, not all patients were virologically suppressed. Specifically, 15 participants (18.9%) had HIV‐1 RNA levels between 50 and 200 copies/mL prior to switching to B/F/TAF. These individuals achieved virological suppression (< 50 copies/mL) by Week 24 of treatment, demonstrating the effectiveness of the regimen in this subpopulation. Among these patients, some exhibited transient viral blips (*n* = 8), while others maintained persistent low‐level viremia (between 50 and 200 copies/mL) prior to suppression (*n* = 7). Most patients were treated with PI‐based ART. The median viral load for this group decreased significantly from 74 copies/mL at baseline to 21 copies/mL at Week 24 (*p* < 0.001). A statistically significant difference was observed between baseline and week 24 HIV viral load (McNemar’s test, *p* ≤ 0.046). In the analysis of effectiveness by subgroup, no statistically significant differences were detected concerning either previous ART regimen or period of diagnosis.

The number of daily tablets each participant required decreased significantly; up to 27.8% (*n* = 22) of the patients reduced the number of tablets taken (*p* ≤ 0.001). There were no significant changes from baseline to Week 24 in the CD4+ lymphocyte count, lipid profile, or glomerular filtration rate.

### 3.3. Safety and AEs at Week 24

AEs were reported in four patients (5.1%), which included diarrhoea, headache, insomnia, and weight gain in one patient each (Table 3). Three patients discontinued treatment due to AEs; none of them were serious or fatal. There were no significant changes from baseline to Week 24 in the incidence of renal AEs.

**Table 3:** Clinical evolution of patients treated with B/F/TAF.

	All patients (*N* = 79)
Follow‐up duration, median (IQR), months	23 (12–33)
Outcomes	
Remain on B/F/TAF, *n* (%)	65 (82.3)
Treatment switch, *n* (%)	5 (6.3)
Before Week 24	1 (1.3)
After Week 24	4 (5.1)
Intolerance	3 (3.8)
Patient decision	1 (1.3)
Withdraw TAF	1 (1.3)
Death, *n* (%)	4 (5.1)
Loss of follow‐up, *n* (%)	5 (6.3)
AEs, *n* (%)	4 (5.1)
Diarrhoea	1 (1.3)
Headache	1 (1.3)
Insomnia	1 (1.3)
Weight gain	1 (1.3)
Time to suspension, median (IQR), months	17 (6–27)
Treatment switch, *n* (%)	
DTG‐RPV	2 (2.5)
DTG‐3TC	2 (2.5)
RAL‐FTC‐TAF	1 (1.3)

Abbreviations: AEs = adverse events, B/F/TAF = bictegravir/emtricitabine/tenofovir alafenamide, DTG‐3TC = dolutegravir/lamivudine, DTG‐RPV = dolutegravir/rilpivirine, IQR = interquartile range, RAL‐FTC‐TAF = raltegravir/emtricitabine/tenofovir alafenamide, TAF = tenofovir alafenamide.

### 3.4. Follow‐Up

Patients were followed for a median of 23 months (interquartile range: 18–30 months) after switching to B/F/TAF. Among the six patients who did not achieve virological suppression, two exhibited VF failure associated with documented suboptimal adherence; no resistance mutations were detected in genotypic tests. Safety outcomes were favourable, with AEs reported in 5.1% (*n* = 4) of patients, and treatment discontinuation occurred in 6.3% (*n* = 5), primarily due to intolerance. In these participants, the median duration of treatment until discontinuation of B/F/TAF was 17 months (IQR 6–27).

### 3.5. Subgroup Analysis: Patients Switching From NNRTI‐Based Regimens

Among the 50 patients who switched from NNRTI‐based regimens, key virologic and immunologic outcomes mirrored those observed in the overall cohort. Virologic suppression at Week 24 was maintained in forty‐eight (96%) of this subgroup, comparable to the total population. Similarly, immune parameters remained stable, endorsing a durable immunologic response. Univariate analysis showed that patients proceeding from the NNRTI group had a significantly higher probability of maintaining treatment, with an odds ratio (OR) of 8.030 and a significant *p* value (*p* ≤ 0.008) in the multivariate logistic regression model. This suggests a statistically robust association with therapy persistence compared to other regimen backgrounds after switching to B/F/TAF. Baseline CD4 count was also significantly associated with the likelihood of maintaining B/F/TAF treatment, although with a small effect size (OR close to 1, *p* ≤ 0.047), while variables like sex, age, baseline viral load, and the number of prior treatment pills were not significant predictors.

**Figure 1:**
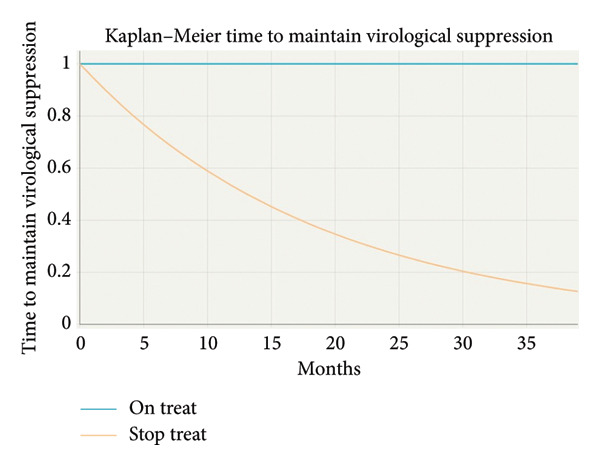
Kaplan–Meier curve showing time to loss of virological suppression over 36 months in patients who remained on treatment versus those who discontinued treatment.

The Kaplan–Meier time to maintain virological suppression analysis (Figure 1) illustrates the proportion of patients maintaining virological suppression over time, comparing the groups “on treatment” and “stop treatment” over a period measured in months. For the stop treatment group, the mean survival time was 18.88 months (95%; 12.17–25.58). In contrast, the on‐treatment group showed a mean survival time of 39 months (95%; 39–39). The nonoverlapping confidence intervals between the groups showed statistically significant differences in survival (*p* ≤ 0.017). The absence of variability in the “on treatment” group reflects complete censoring, meaning all subjects remained event‐free until the study’s maximum observation time of 39 months. In the analysis of effectiveness by subgroup, no statistically significant differences were detected concerning either previous ART regimen or period of diagnosis.

## 4. Discussion

This single‐centre study demonstrates that switching virologically suppressed individuals from non‐INSTI‐based ART regimens to B/F/TAF is both effective and safe, with virological suppression rates of 92.4% (ITT) and 96.1% (per‐protocol), alongside a low incidence of AEs (5.1%) and treatment discontinuation (6.3%) [6, 11–14]. Our findings are consistent with other retrospective studies evaluating the B/F/TAF switch (Table 4) [6, 11–14, 16].

**Table 4:** Retrospective studies evaluating the efficacy and safety of switching to B/F/TAF.

Study	Population, *n*	Age, years (range)	Males, *n* (%)	Previous ART, *n* (%)		B/F/TAF duration (range)	Effectiveness	
Lazzaro et al. 2021 [15]	147	57 (49–61)	104 (70.7)	PI	97 (66.0)	12 months	% pts with viral load < 37 copies/mL at Week 48	93 (OT)
				INSTI	23 (15.6)			
				NNRTI	20 (13.6)			
				PI + INSTI	7 (4.9)			
				No INSTI	117 (79.6)			

Ambrosioni et al. 2022 [16]	1584 (pretreated 1371; 1046 included)	44 (36–53)^a^	1185 (86)^a^	INSTI	721 (69)	16.4 months (7.6–21.3)^a^	% pts with viral load < 50 copies/mL at Week 24^a^	94% (OT) 83% (ITT)
				NNRTI	207 (20)			
				PI	93 (8)			
				Other	25 (3)		% pts with viral load < 50 copies/mL at Week 48^a^	93% (OT) 78% (ITT)
				No INSTI	325 (23)			

Rolle et al. 2021 [14]	115 (pretreated 87)	43 ± 11.5^b^	82 (71.3)^b^	INSTI	70 (80.4)	8.7 ± 6.2 months^b^	% pts with viral load < 50 copies/mL at Week 24	94.1 (OT)
				NNRTI	9 (10.5)			
				PI	5 (5.7)			
				INSTI + PI	3 (3.4)			
				No INSTI	17 (19.5)			

Molina et al. 2021 [6]	175	39 (32–49)	171 (97.7)	INSTI	74 (42.3)	13 months (10.5–15.8)	% pts with viral load < 50 copies/mL during treatment	94.9 (ITT)
				PI	65 (37.1)			
				NNRTI	34 (19.4)			
				Other	2 (1.2)			
				No INSTI	101 (57.7)			

Rolle et al. 2021 [16]	350	57 (50–81)	281 (80)	INSTI	196 (56)	12 months	% pts with viral load < 50 copies/mL at Week 48	94 (OT)
				NNRTI	80 (23)			
				PI	45 (13)			
				Other	21 (6)			
				INSTI + PI	8 (2)			
				No INSTI	125 (35.7)			

Hagins et al. [8]	495	49 (18–79)	229 (69)	INSTI PI NNRTI	202 (61) 30 (9) 100 (30)	61 weeks	% HIV‐1 RNA, 50 copies/mL at 48 weeks	97% (ITT)

BIKSWITCH Perez‐Calvo et al.	79	54 (43–59)	66 (80.5)	NNRTI	50 (63.3)	23 months (12–33)	% pts with viral load < 50 copies/mL at Week 24	96.1 (OT) 92.4 (ITT)
				PI	28 (35.4)			
				PI + NNRTI	1 (1.3)			

Abbreviations: ART = antiretroviral treatment, B/F/TAF = bictegravir/emtricitabine/tenofovir alafenamide, INSTI = integrase strand transfer inhibitors, ITT = intent to treat, NNRTI = non‐nucleoside reverse transcriptase inhibitor, OT = on‐treatment, PI = protease inhibitor, pts = patients.

^a^Only includes patients who were pretreated.

^b^Includes all patients.

The demographic and clinical profile of our cohort—median age in the mid‐50s and predominance of male participants—aligns with other real‐world cohorts [6, 11–14]. Notably, unlike most studies including patients switching from INSTI regimens, our analysis focuses on an underrepresented population switching from non‐INSTI therapies, reflecting the findings of Molina et al. [6].

We observed a significant increase in the proportion of participants achieving HIV‐1 RNA < 50 copies/mL postswitch, with maintained suppression in those with detectable baseline viral loads, affirming B/F/TAF’s efficacy [6, 11–14]. The VF rate (2.6%) in our cohort parallels rates reported in clinical trials by Daar et al. and Sax et al., as well as real‐world data from Molina et al. [6]. Pozniak et al. [9] similarly indicates VF rates below 2% at 48 weeks in trials.

Torralba et al.’s [17] multicentre cohort study corroborates these findings, demonstrating B/F/TAF’s effectiveness, safety, and durability. A meta‐analysis by Chivite et al. [18] further supports its low discontinuation and high viral suppression rates in both treatment‐naïve and experienced populations.

Our demonstration of a strong association between exposure to prior NNRTI‐based regimens and persistence on B/F/TAF (OR 8.03) agrees with Italian BICSTaR cohort real‐world data from Esser et al. [19], who reported excellent treatment persistence and immunologic outcomes.

Although baseline viral load predicts VF in other cohorts, all patients in our study with baseline viral loads between 50 and 200 copies/mL suppressed virus to undetectable levels by Week 24. Molina et al.’s [6] data reflect similar but somewhat lower suppression rates. Collectively, our results, consistent with clinical trials and observational cohorts, affirm B/F/TAF’s robust genetic barrier and appropriate use in patients lacking documented resistance [6, 11–14].

Metabolic safety is supported by Molina et al. [6] and Rolle et al. [14] observations of stable lipid and renal parameters after switching. While some studies report lipid profile improvements postswitch [8, 13, 14], this was less evident in our cohort, likely due to a low proportion of abacavir users (6.3%).

AEs leading to discontinuation were infrequent and mild. Gutiérrez‐Lorenzo et al. [12] found similar discontinuation rates under 5% of the time, and Lazzaro et al. [13] reported predominantly mild adverse effects. Our study similarly noted headache and weight gain as the most frequent side effects without serious events [6, 11–13]. Treatment changes were low (6.3%) and due to AEs, patient decision, and TAF discontinuation because of renal safety concerns.

Drug–drug interaction reduction motivated many switches, consistent with other series highlighting polypharmacy risks in older patients. Unlike prior cohorts [6, 11, 13, 14], simplification was less common in ours, although 27.8% of patients reduced tablet counts postswitch, echoing Ambrosioni et al.’s [11] findings on improved adherence after pill burden reduction.

### 4.1. Limitations

Our study has several limitations inherent to its retrospective and single‐centre design. There is a risk of underreporting epidemiological data and baseline characteristics, common in retrospective analyses. Although missing data for the primary outcome was minimal, up to 24% of secondary variables were missing, which may affect some results. Data collection by a single researcher ensured some consistency.

The sample size is relatively small, limiting generalisability, although it is comparable to other published cohorts. Importantly, this study focuses on patients switching from NNRTI‐based regimens to B/F/TAF, an underrepresented subgroup in larger cohorts.

Pre‐existing resistance was not systematically assessed, and AEs were only reliably recorded if they led to treatment discontinuation, potentially skewing safety findings. Lack of data on lipid‐lowering therapy use further complicates interpretation of metabolic changes.

Resistance testing was not routinely performed, limiting understanding of VF mechanisms. Weight was inconsistently recorded, constraining metabolic safety assessment.

VF was attributed to suboptimal adherence based on indirect evidence, but adherence data were not systematically collected. The potential impact of drug interactions, pharmacokinetic issues, and psychosocial factors on adherence was not addressed.

## 5. Conclusions

Switching from INSTI‐based antiretroviral therapy to B/F/TAF is both safe and highly effective for people living with HIV. Our findings demonstrate that this switch not only maintains, but in many cases improves, virologic control, as evidenced by a significant increase in the proportion of participants achieving viral suppression at 24 weeks compared to their baseline regimen. Furthermore, B/F/TAF exhibits an excellent safety profile, with a low rate of AEs and very few treatment discontinuations attributed to side effects. These results underscore the suitability of B/F/TAF as a robust and well‐tolerated option for individuals transitioning from non‐INSTI‐based therapies.

## Ethics Statement

This study was conducted in accordance with the Declaration of Helsinki. The study protocol was approved by the Ethics Committee for Drug Research of the Alicante University General Hospital Department, Alicante, Spain, on December 22nd, 2022, with reference number 2022‐130. On January 9th, 2023, the Research Ethics and Integrity Committee of the Vice President for Research of Miguel Hernández de Elche University, Alicante, Spain, approved the Code of Responsible Research (COIR). Ethics approvals included permission to access patient clinical records.

## Consent

A waiver of informed consent to use retrospectively collected data that were de‐identified.

## Disclosure

All authors approved the manuscript.

## Conflicts of Interest

The authors declare no conflicts of interest.

## Author Contributions

All authors contributed equally to this study regarding design, enrolment of patients, performance of some of the analyses, and the preparation of the manuscript.

## Funding

This study, the development of the manuscript, and the open access publishing fee were supported by Gilead Sciences Inc.

## Data Availability

The datasets used and/or analysed during the current study are available from the corresponding author upon reasonable request.
